# Organic Poisons

**Published:** 1873-12-01

**Authors:** E. W. Gray

**Affiliations:** Bloomington, Ill.


### ORGANIC POISONS.

AN ESSAY READ BEFORE THE McLEAN COUNTY MEDICAL SOCIETY, OCTOBER 6, 1873, BY E. W. GRAY, M.D., BLOOMINGTON, ILL.

Until the microscope had made its revelations it was scarcely possible for us to have any adequate conception of the almost infinite variety and abundance of the living forms that swarm unseen in earth and air and water. We had heard of the poetic insect that floats in the sunbeam, or gambols in the dew-drop; but that countless millions of living beings actually fill the air, and fill the ocean—that we constantly breathe them and eat them and drink them—that they should come to live in our eyes and ears and mouths and stomachs and bowels and livers—that they should come to infest the skin and mucous membrane in all their ramifications, was too much for our ante-microscopic imagination. Nevertheless, such is the fact. The wonder is that they do not destroy us outright; that we can live at all amid such a cloud and swarm of infinitesimal vampires, ever eager to prey upon us, and eat out our lives.

That some of the exanthems are occasioned by the invasions and depredations of some of these animalculæ is a fact now well ascertained. To effect a cure, the physician needs only to apply a poison and kill them. In addition to these myriads of animal organisms, there are also equal numbers of living vegetable forms—spores and fungi—covering the earth and floating in the air and swarming in the water. Some of these, admitted to the blood, rapidly develop and multiply and engender disease.

The inorganic poisons once taken into the system, emerge from it sooner or later, unaltered; or entering into more permanent chemical union with the constituent tissues of particular organs, interfere in various ways with their functions. But the organic poisons act differently. Having gained admission to the blood or other fluids of the system they rapidly vitiate them; or, becoming a source of irritation, destroy the vital functions. In the blood they find the requisite conditions: the degree of warmth and the nutriment necessary for their rapid development and sustenance. Being inaccessible by any means yet discovered, they cannot be destroyed, and so run their course, and finally perish, in accordance with the law of their being.

But what is the evidence that there are organic poisons?

1. In the first place, if the hypothesis—for as yet it is little more than an hypothesis—accords with known facts, and accounts for various symptoms of several diseases more satisfactorily than can otherwise be done, we must entertain it. Now, we know that these infinitesimal organisms do abound in infinite profusion; and that they do feed upon and consume the fluids of the living body; and we may infer that if they could but gain admission through the membrane everywhere interposed between these fluids and the external world, they would revel in the rich luxuriance thus offered to their voracity.

2. We know that some diseases are actually produced by the depredation of certain animalculæ, as psora or scabies. Psoriasis and the various forms of porrigo are so similar in symptoms, and so subject to like treatment, that it is highly probable that they are all produced by like causes. We know, also, that vibriones exist in abundance in the pus of internal abscesses, whether they serve as factors in producing and maintaining the disease or not. I instance, also, the well-known presence of entozoa, which in one form or another have been found infesting the alimentary canal and other cavities, and producing disease. These facts, taken in connection with what we now know of the existence of countless varieties of microscopic organisms, of whose disease-producing power we are as yet ignorant, or but partially informed, render it extremely probable that most of our fevers, so called, and especially endemic, epidemic, and contagious diseases, are produced by the presence of some of these infusoria, and their interference with the vital functions.

3. Again, we know that all organic life has its period of existence. This is true, not only of vegetable life, but of all animal life. It is generated. It incubates. It lives. It dies. This is the short history of all organic beings, animal and vegetable. There are certain diseases which have periods corresponding very closely to this order of life. They are generated. They incubate. They run their course and die out; but always disturbing the functions of life; sometimes killing the patient. The *modus operandi* of quinine in the cure of malarial fever has never been satisfactorily explained. Dr. Hyatt, in a paper read before the Ohio State Medical Society a year or two ago, attempted an explanation. He supposed that from some cause there occurs a deficiency of a certain alkaline ingredient of the blood; that quinine for a time supplies this want, and suspends the disease without destroying the producing cause. Hence, when the quinine is exhausted the disease returns. He thinks if we would remove the cause and permanently cure the disease we must use arsenic.

This theory entirely overlooks the significant fact that the disease is wont to return at regularly recurring periods, and only asserts that by one catalysis or chemical change quinine supplies a given want, and that arsenic, by another, destroys the poison which produces the disease. It would, I think, be easier to account for the facts accompanying the cure of intermittent fever by supposing that the disease is occasioned by some organic poison, and that the quinine and the arsenic operate to destroy the zymosis which has been established; and, if continued, will at last destroy any zymosis which may be incubating in the system. There are, doubtless, many other agents which would accomplish the same result, if we but knew them and could apply them. In syphilis, Dr. Hyatt admits an organic poison, which mercury, taken at the proper time, destroys, and thus effects a cure.

In *Braithwaite*, for 1872, a large number of cases of typhoid fever—seventy or more—are reported as having been treated successfully with sulphurous acid, there not being a single failure to cure. Such remarkable success, I grant, is calculated to awaken suspicion as to the genuineness of the report. But it must be admitted that the reporter is in pretty good company; and I instance the account of these cases only as indicating the possible results the healing-art may attain when we come to know more of the causes of disease. If typhoid fever is produced by some organic poison, and sulphuric acid, or carbolic acid, or any other known agent, is competent to destroy it, why not cure every case, if called to the patient before the vital powers have been fatally impaired?

In the last number of *Braithwaite* a case is given of an intelligent young farmer, who, in making a post-morten examination of an animal that had died of some strange disease, had his hand scratched, and was poisoned. Some dogs and hogs that ate of the diseased animal soon died. Alarming symptoms came on, and it was soon discovered that the young man's life was threatened. Large doses of quinine were given and he rapidly recovered. Now, if we suppose, what is highly probable, that some organic poison had entered the blood through the scratch upon his hand, which, like barm in the manufacture of beer, rapidly reproduced itself, and as rapidly vitiated the blood, we can understand that he would have been cured by any anti-zymotic that would destroy the life of the poison.

In a late number of *The Clinic* is the report of a discussion on the subject of hay-fever. Twelve different remedies were suggested as having been tried with advantage; but it was evidently felt that none of them could be relied on. There seemed to be a kind of general agreement or consent that it is a *nervous affection*. Dr. Elliotson long ago suggested, and, I may add, with characteristic discrimination, that disinfectants would probably be useful, and recommended the chloride of lime and of soda—remedies which were afterwards found to be eminently useful in curing the disease.

Some time ago, Dr. J. L. White, of this city, having a case of this disease, and suspecting it might be produced by an organic poison, directed the local application of a strong solution of quinine, and had the satisfaction of witnessing almost immediate and very decided relief, and in a few days a perfect cure.

According to the latest knowledge on the subject, diphtheria is a miasmatic, contagious disease, during which masses of fermenting parasites are formed; and these parasites carry with them the destroying element, which they convey through the blood and lymph courses more or less rapidly to the whole organism. They can only be destroyed by disinfecting or anti-zymotic remedies. According to Max, Jaffe and Oertel, who have written largely on the subject, sulphur and carbolic acid are the most efficacious remedies that can be employed. See *The Clinic* for September.

Many other cases could be given of diseases which, either in their symptoms or in their successful treatment, demonstrate the possible existence of organic poisons as prolific causes of disease, and the conviction that this is so, daily grows stronger in the minds of medical men, as is abundantly evinced in the current literature of the profession, and in the growing use of disinfectants and antizymotics generally.

All substances, animal and vegetable, exist and grow by the generation, reproduction and aggregation of cells. This is true alike of normal and abnormal substances in the animal organism. In their legitimate growth and decay we have the regular process of nature—the regeneration and "wear and tear" of healthy tissue. In their illegitimate growth we have cancer, and all tumors whose processes of life interfere with, and sometimes overmaster and destroy, the affected parts. Thus, all the disease and suffering and death which they produce are but the products of organic but misplaced life—one organism sustaining itself at the expense of another. It is true, we do not yet know in what subtile, perverting power this type of life originates; but we do know that to preserve one life we must destroy another; and the essential difficulty in the cure of such diseases is not unlike that of the farmer who finds it impossible "to gather up the tares without rooting up the wheat also."

If these views be correct—if it is really life that so often produces death—we shall need to abandon many old remedies and depletory measures, and seek for remedies whose life-destroying power is specific, and suited to the several cases that exist. Fortunately, what destroys one life promotes another. Everywhere life survives by producing death; and if we could always know how to protect and properly direct the forces of the better life, it would not be necessary that it should ever yield to the worse—the lower life. While the gastric juice, the bile and intestinal fluids operate to destroy most of the living beings that find their way into the alimentary canal, they are not only harmless, but necessary to the organism which they serve; and we may hope that other agents will be discovered which are innocent as to the human system, but destructive of the parasites and the thousands of microscopic organisms which threaten our health, and which, in the instinctive, eager pursuit of their own life, urge on the work of our destruction.

